# The Influence of Intraocular Lenses (IOLs) on the Properties of Filters Protecting Human Eyes against Optical Radiation in the Work Environment

**DOI:** 10.3390/ijerph19031793

**Published:** 2022-02-04

**Authors:** Grzegorz Owczarek, Joanna Szkudlarek, Natalia Skuza

**Affiliations:** 1 Department of Personal Protective Equipment, Central Institute for Labour Protection—National Research Institute, 48 Wierzbowa Street, 90-133 Lodz, Poland; joszk@ciop.lodz.pl; 2 Department of Ophthalmology and Vision Rehabilitation, Military Medical Academy Memorial Teaching Hospital of the Medical University of Lodz—Central Veteran’ Hospital, 113 Zeromskiego Street, 90-549 Lodz, Poland; natalia.mloczkowska@onet.eu

**Keywords:** luminous transmittance, optical protective filters, intraocular lenses IOLs, harmful optical radiation

## Abstract

Under the specific illumination conditions of many workplaces, e.g., in the metallurgical industry, decreased lighting may impair workers’ vision and, as a result, their productivity. Spectrophotometric tests of two types of protective optical filters (welding filters and infrared protection filters), two types of intraocular lenses (IOLs with and without yellow chromophore), and filter-IOL systems were carried out. In spectrophotometric studies, the spectral characteristics of transmission and the coefficients for the assessment of light transmission were determined. This study explores the relationship between the eye protection levels offered by filters and the use of intraocular lenses (IOLs), and especially those containing a yellow chromophore which may lower the luminous transmittance of protective filters. In our previous works, we studied a large number of optical protective filters and many factors influencing their performance. A review of the literature has shown the absence of prior research on the subject. For this purpose, transmittance reduction factors were defined for the evaluation of the filter-IOL system. The spectral characteristics of luminous transmittance for the tested IOLs indicate a significant decrease of transmittance for those with yellow chromophore within the range up to approx. 475 nm, as compared to IOLs without chromophore. The main objective of this study was to determine whether people with IOLs need different protective filters against harmful optical radiation as well as whether IOLs may change the required category of protective filters. The key finding is that while the use of IOLs in conjunction with protective filters does change the light transmission coefficient, it does not affect the filter protection levels. The transmittance reduction factors were similar (0.95 to 0.99 relative units) for all filter-IOL systems irrespective of the presence or absence of yellow chromophore. It must be said clearly that, in reference to the requirements specified in the standards, IOLs did not affect the filter protection levels. This means that the quality of vision did not change significantly when using the analyzed filters and IOLs.

## 1. Introduction

The objective of the study was to examine changes in vision quality in situations where individuals with intraocular lens implants are obligated to use protective filters. Given that eye protection devices are routinely used in certain workplaces, it was necessary to investigate the effects of IOLs on the protective performance of those devices (filters). This paper presents a method for determining the luminous transmittance of systems consisting of protective optical filters and intraocular lenses (IOLs), using standard and real illuminants with different spectral power distributions. Spectral characteristics were measured for two types of IOLs (clear and yellow—with yellow chromophore) and for two types of protective optical filters (welding and IR filters—protecting against infrared radiation), which significantly differed in optical density. A light transmittance reduction factor for the filter-IOL systems was defined. The methodology presented in this paper and the obtained results can be used to determine the effects of IOLs on the classification of protective optical filters for people with implanted IOLs. The paper described consecutive methodological steps in the evaluation of the protective performance of the filter-IOL system.

There is some clinical and investigative evidence that blue light filtering IOLs (yellow IOLs) may reduce the risk of age-related macular degeneration [1]. The literature is not conclusive in terms of the influence of IOLs on contrast sensitivity and color vision [2,3]. Small differences in visual function were noted by Wirtitsch et al. in the case of tinted lenses [4]. In turn, Eberhard et al. [3] did not arrive at a clear answer as to the effects of two types of lenses on preserving visual function. However, according to several authors, contrast and color sensitivity decrease under mesopic conditions [5,6,7,8]. Mainster reported that yellow-tinted IOLs could have a negative influence on scotopic and mesopic contrast sensitivity due to the Purkinje shift, since blue light is much more important for scotopic than for photopic vision [9,10]. In a work by Li et al., blue-light-filtering IOLs reduced scotopic sensitivity by 14–21%, depending on dioptric power [11]. Ao et al. [6] found that there was a statistically significant difference in partial error scores in the green to blue-green color band under mesopic, but not photopic, conditions. The literature is clear on mesopic and scotopic contrast sensitivity in people with IOLs. This concerns many professionals, e.g., steelworkers and welders, who need to use protective filters in the workplace. To date, no studies have examined the influence of clear or yellow IOLs on the vision of users of optical filters. Visual function (including color recognition, identification of objects in the surroundings, control of technological processes, etc.) during use of optical filters depends on the spectral characteristics of the filters in the visible range. So far, the effectiveness of protective optical filters has been determined with regard to the protective filter-eye system. If the natural lens is replaced by an IOL, the level of harmful optical radiation blocked can change, and certain eye functions that are important while performing tasks in the work environment may be modified since visual stimuli may be received differently by persons after cataract surgery or refractive correction with clear or yellow IOL implantation, especially under low light conditions (characteristic of certain work stations). The objective of the work was to determine whether people with IOLs will need different levels of protection while using optical filters against harmful optical radiation as well as whether IOLs can change the category of protective filters required by them.

## 2. Materials and Methods

The study involved two acrylic and hydrophobic intraocular lenses: Alcon SN6AT3 and Artis PL E. The Alcon lens has a yellow chromophore (blue light filter) absorbing wavelengths of up to approx. 475 nm to prevent the harmful effects of radiation in this range on the retina. The IOLs were used in conjunction with welding and infrared (IR) filters characterized by different degrees of optical density to visible radiation; these included welding filters [12] with shade numbers: 2 (sample 3), 5 (sample 4), 8 (sample 5), 9 (sample 6), 10 (sample 7), and 12 (sample 8) as well as IR filters [13] with shade numbers: 4–3 (sample 9), 4–4 (sample 10), 4–5 (samples 11 and 12), and 4–7 (sample 13).

The spectral characteristics of the tested samples (intraocular lenses, welding filters, and IR filters) were determined using a CARY 5000 Varian spectrophotometer (Australia). In the sample compartment, each filter and IOL was placed so that the measuring beam would fall perpendicularly to its surface and then directly reach the photodetector. 

A mathematical method is hereby proposed for determining the spectral characteristics of filter-IOL systems to eliminate the decrease in transmission levels associated with spectrophotometric measurement in the sample compartment. In this method, the spectral characteristics of the filter (*τ*(*λ*)_F_) and the IOL (*τ*(*λ*)_IOL_) are calculated separately and are then combined to compute the filter-IOL transmission coefficient (*τ*(*λ*)_F+IOL_). The spectral transmission coefficients of the IOLs and filters comprising filter-IOL systems are expressed by Formulas (1) and (2).

(1) $$\tau {\left(\lambda \right)}_{\mathrm{IOL}}=\frac{Q_{1}(\lambda )}{Q_{0}(\lambda )}$$

(2) $$\tau {\left(\lambda \right)}_{F}=\frac{Q_{2}(\lambda )}{Q_{1}(\lambda )}$$

where:

*Q*_0_(*λ*)—light beam incident on the filter,

*Q*_1_(*λ*)—light beam passing through the filter, 

*Q*_2_(*λ*)—light beam passing through the filter-IOL system.

A formula for the luminous transmittance of filter-IOL systems is obtained from Formulas (1) and (2), and is expressed as follows.

(3) $$\tau {\left(\lambda \right)}_{F+\mathrm{IOL}}=\frac{Q_{2}(\lambda )}{Q_{0}(\lambda )}=\frac{\tau {\left(\lambda \right)}_{F}\cdot Q_{1}(\lambda )}{Q_{0}(\lambda )}=\tau {\left(\lambda \right)}_{F}\cdot \tau {\left(\lambda \right)}_{\mathrm{IOL}}$$

As can be seen from Formula (3), the luminous transmittance of the filter-IOL system (*τ*(*λ*)_F+IOL_) is a product of the spectral transmittance of the filter (τ(λ)_F_) and that of the IOL (τ(λ)_IOL_). Spectral characteristics were used to determine luminous transmittance in accordance with Formula (4) below [14].

(4) $$\tau_{\nu }=\frac{\int_{380\;\mathrm{nm}}^{780\;\mathrm{nm}}\tau (\lambda )\cdot S\left(\lambda \right)\cdot V\left(\lambda \right)d\lambda }{\int_{380\;\mathrm{nm}}^{780\;\mathrm{nm}}S\left(\lambda \right)\cdot V\left(\lambda \right)d\lambda }$$

where:

*τ* (*λ*)—spectral transmittance,

*S*(*λ*)—spectral power distribution of the illuminant,

*V*(*λ*)—spectral sensitivity function of the average human eye to daylight.

**Figure 1:**
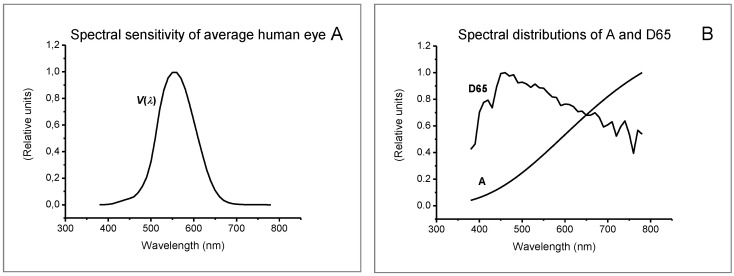
Spectral distributions of the standard weight functions used to determine luminous transmittance: (**A**) spectral sensitivity function of the average human eye to daylight [15], (**B**) spectral power distribution for standard illuminants A and D65 [16].

**Figure 2:**
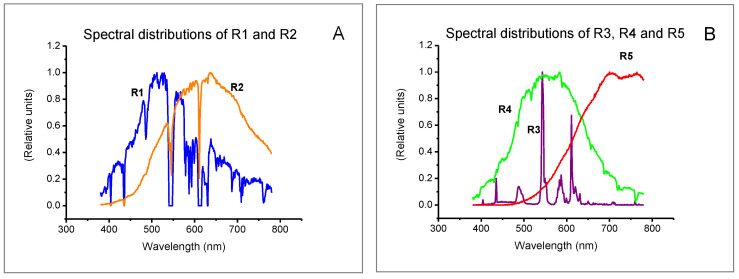
Spectral power distributions of illuminants determined under real conditions: (**A**) illuminants R1 and R2, (**B**) illuminants R3, R4, and R5.

Luminous transmittance was determined from Formula (4) for the spectral sensitivity function of the average human eye to daylight and the spectral power distribution of standard illuminants (A, D65) as well as illuminants corresponding to the following real conditions: R1—natural light from a cloudy sky, R2—room lit by halogen lamps (Osram, 59 W/12 V), R3—room lit by fluorescent lamps (Philips TLD 58/840), R4—room lit by LED lamps (SMD 3630, 5.9 W/11 V/350 lm, 3000 K), and R5—room lit by radiation emitted from a laboratory furnace operating at a temperature of 1000°C. The real illuminants were supposed to mimic actual working conditions. Spectral power distributions were determined using an HR 2000+ spectroradiometer (Ocean Optics, USA) equipped with an optical fiber and an optical radiation diffuser. Figure 1 presents the spectral distributions of the standard weight functions used to determine luminous transmittance, i.e., the spectral sensitivity function of the average human eye to daylight and the spectral power distribution for illuminant D65. Figure 2 presents the spectral distributions of illuminants determined under real conditions (from R1 to R5).

Seven values of luminous transmittance were determined for each of the samples tested: intraocular lenses, welding filters, and IR filters (*τ_v/_*_day*/i*_ with *i* representing the illuminant included in the calculation, that is, D65, A, R1, R2, R3, R4, or R5). Luminous transmittance was also determined for filter-IOL systems.

Subsequently, relative changes in luminous transmittance for welding filters and IR filters, as well as filter-IOL systems were determined. Relative changes in luminous transmittance were determined from Formula (5).

(5) $$\Delta \tau_{v/i}=\frac{\tau_{v/\mathrm{day}/i/F}-\tau_{v/\mathrm{day}/i/F+\mathrm{IOL}}}{\tau_{v/\mathrm{day}/i/F}}$$

where:

*i*—symbol of the illuminant included in the calculation (*i* = D65, A, R1, R2, R3, R4, R5),

*τ_v/_*_day*/i/*F_—luminous transmittance of the filter for illuminant *i*,

*τ_v/_*_day*/i/*F+IOL_*—*luminous transmittance of the filter–IOL system for illuminant *i*.

If a relative change in luminous transmittance is expressed as a non-percent value, Formula (5) can be used to define an equation for comparing the luminous transmittance of a filter and that of the filter-IOL system:

(6) $$\tau_{v/day/i/F+\mathrm{IOL}}=\tau_{v/\mathrm{day}/i/F}\cdot (1-\Delta \tau_{v/i})$$

where:

*i*—symbol of illuminant included in the calculation (*i* = D65, A, R1, R2, R3, R4, R5),

*τ_v/_*_day*/i/*F_—luminous transmittance of the filter for illuminant *i*, 

*τ_v/_*_day*/i/*F+IOL_*—*luminous transmittance of the filter-IOL system for illuminant *i*.

The factor (1—Δ *τ_v/i_*) in Formula (6) determines the ratio of the luminous transmittance of the filter-IOL system to that of the filter itself. This factor was defined as the transmittance reduction factor of the filter-IOL system:

(7) $$\eta =(1-\Delta \tau_{v/i})$$

where:

∆*τ_v/i_—*relative change in luminous transmittance determined for the filter and the filter-IOL system.

## 3. Results

Research was conducted according to the following scheme. First, spectral transmittance characteristics were determined for all tested samples (IOLs, welding filters, and IR filters).

Those data were used to calculate luminous transmittance of the tested samples and filter-IOL system, using standard (A, D65) and real (R1 to R5) illuminants. In addition, relative changes in luminous transmittance were computed for filter-IOL systems and filters alone. Finally, the transmittance reduction factor (see Formula (5)) was determined for filter-IOL systems.

### 3.1. Spectral Characteristics of Tested Samples

**Figure 3:**
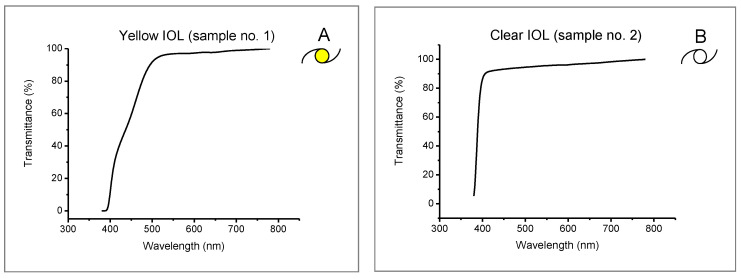
Spectral characteristics of intraocular lenses: (**A**) yellow IOL—with yellow chromophore (sample 1); (**B**) clear IOL—without yellow chromophore (sample 2).

**Figure 4:**
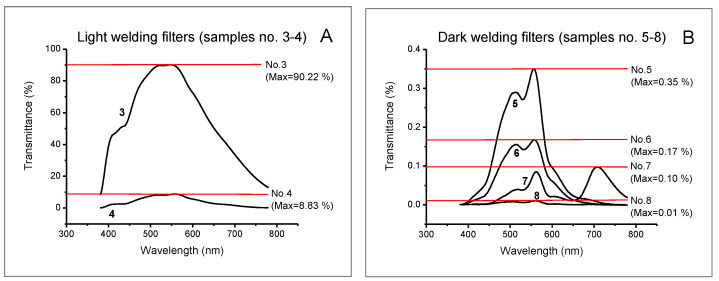
Spectral characteristics of welding filters: (**A**) light welding filters (samples no. 3–4); (**B**) dark welding filters (samples no. 5–8).

**Figure 5:**
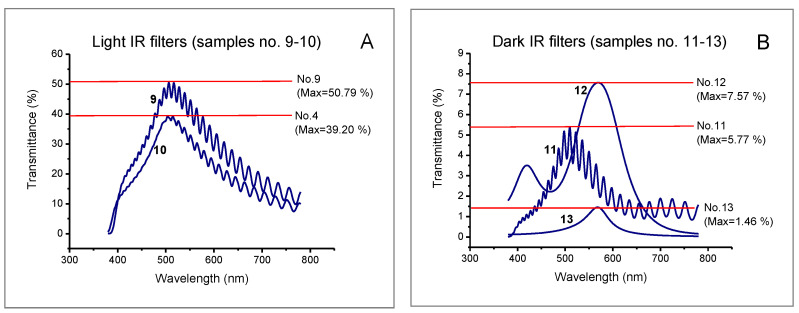
Spectral characteristics of IR filters: (**A**) light IR filters (samples no. 9–10); (**B**) dark IR filters (samples no. 11–13).

The spectral characteristics of all tested samples (intraocular lenses, welding filters, and IR filters) are presented in Figure 3, Figure 4 and Figure 5.

The graphs presented in Figure 3 show the differences in transmitting blue light for yellow IOL. The spectral characteristics of this lens are very similar to a typical blue-blocker spectacle lens.

Welding filters can differ significantly in the transmission of light. Depending on the welding process, and thus on the intensity of the emitted welding radiation, light or dark welding filters are used. Our research included two light (samples no. 3–5) and four dark (samples no. 5–8) welding filters. The maximum spectral transmittance values for light welding filters are 8.83% and 90.22%. For dark welding filters the maximum values of the spectral transmittance are between 0.01% and 0.35%.

Filters designed to protect the eyes against infrared radiation also significantly differ in light transmission. It depends on the intensity of radiation emitted during technological processes. Two light IR filters (samples no. 9–10) and three dark IR filters (samples no. 11–13) were selected for the tests. The maximum spectral light transmittance for the selected filters ranged from 1.46% (dark IR filter, sample no. 13) to 50.79% (light IR filter, sample no. 9).

### 3.2. Luminous Transmittance

Table 1, Table 2 and Table 3 show the results of the calculated values of luminous transmittance of IOLs (see Table 1), welding filters and welding filter-IOL systems (see Table 2), IR filters and IR filter-IOL systems (see Table 3).

### 3.3. Relative Changes in Luminous Transmittance and Transmittance Reduction Factor Determined for Welding Filter and Welding Filter-IOL Systems

Using Formula (5), the values of relative changes in luminous transmittance (see Formula (5)) determined for welding filters and welding filter-IOL systems were calculated. Table 4 and Table 5 show the results of relative changes in luminous transmittance for IOLs with and without yellow chromophore. Using Formula (7), the values of transmittance reduction factor determined for welding filters and welding filter-IOL systems were calculated. Table 6 and Table 7 show the results of transmittance reduction factors for IOLs with and without yellow chromophore.

### 3.4. Relative Changes in Luminous Transmittance and Transmittance Reduction Factor Determined for IR Filter and IR Filter-IOL Systems

Using Formula (5), the values of relative changes in luminous transmittance (see Formula (5)) determined for IR filters and IR filter-IOL systems were calculated. Table 8 and Table 9 show the results of relative changes in luminous transmittance for IOLs with and without yellow chromophore. Using Formula (7), the values of transmittance reduction factor determined for IR filters and IR filter-IOL systems were calculated. Table 10 and Table 11 show the results of transmittance reduction factors for IOLs with and without yellow chromophore.

## 4. Discussion

Plots of the spectral characteristics of luminous transmittance obtained for the tested IOLs (see Figure 1) indicate a significant decrease of transmittance for those with yellow chromophore within the range up to approx. 475 nm, as compared to IOLs without chromophore. However, this does not mean that luminous transmittance determined for the entire visible light range from 380 to 780 nm was lower for yellow IOLs vs. clear IOLs. As can be seen from Table 1, irrespective of the illuminant distribution, those coefficients differed only slightly except for illuminant R1 (natural light from a cloudy sky), where the difference (Δ *τ_v_*_/IOL_ = *τ_v_*_/IOL/clear_ − *τ_v_*_/IOL/yellow_) was 9.29%. That means that under low-intensity daylight, yellow IOL transmitted much less light. In all the other cases (illuminants D65, A, and R2–R5), the luminous transmittance for IOLs with chromophore was slightly higher than for clear IOLs, with the differences ranging from 0.15% (for R3) to 2.09% (for R5). Relative changes in luminous transmittance for clear and yellow IOLs ranged from 2.13% (for R5) to 10.78% (for R1).

The luminous transmittance coefficients determined for two types of IOLs under standard (D65; A) and real (R1–R4) illuminants indicate that the amount of light reaching the retina (vision quality) slightly declined for the users of IOLs with yellow chromophore working under low-intensity daylight (R1—cloudy day). According to the literature, mesopic and scotopic conditions may affect vision in individuals with IOLs. Therefore, the study involved protective filters used in the welding and foundry industry, which may lead to mesopic or scotopic vision.

Plots of the spectral characteristics of luminous transmittance obtained for the tested welding filters (see Figure 2) show that irrespective of filter shade number, maximum transmission occurred from approx. 520 to 550 nm. The luminous transmittance for welding filters (see Table 2) did not differ significantly for illuminants D65, A, and R1–R4. In the case of the distribution of illuminant R5 (room lit by radiation emitted from a laboratory furnace), the luminous transmittance coefficients for all tested welding filters were lower than for the other illuminants. In the case of the darkest filter tested (sample 8), the difference in luminous transmittance coefficients determined for illuminants D65 (*τ_v/_*_day/D65_ = 0.0060%) and R5 (*τ_v/_*_day/R5_ = 0.0057%) amounted to as little as 0.0003%. However, the difference was significant as the filter was already very dark. The relative change in the luminous transmittance coefficient obtained for that filter using the standard illuminant D65 was as much as 31.7% as compared to R5. In turn, as far as the least dark welding filter is concerned (sample 3), the difference in luminous transmittance between D65 and R5 was 7.93%. The relative change in the luminous transmittance coefficient for that filter, measured for the standard illuminant D65 vs. R5, was 9.66%. In the case of the other tested welding filters (samples 4–7), those changes were 15.2%, 31.0%, 28.3%, and 15.2%. Importantly, they were not affected by the IOL type (yellow or clear). Nevertheless, the luminous transmittance of IOLs with chromophore tended to be slightly higher, as already mentioned.

Relative changes in the luminous transmittance for welding filters and welding filter-IOL systems were calculated on the basis of data from Table 2 (see Equation (5)). Table 4 and Table 5 give the results of those calculations for IOLs with and without yellow chromophore, respectively. As can be seen from those tables, relative changes in the luminous transmittance did not depend on how dark the filter was. Rather small differences were found for different illuminant distributions, with lower values obtained for illuminant R5.

As can be seen from Table 6 and Table 7, the calculated transmittance reduction factors were similar (0.95 to 0.99 relative units) for all welding filter-IOL systems irrespective of the presence or absence of yellow chromophore. This indicates that the quality of vision through the analyzed welding filters will not change for the analyzed cases.

Plots of the spectral characteristics of luminous transmittance obtained for the tested IR filters (see Figure 3) show that irrespective of filter shade number, maximum transmission was found between approx. 500 and 550 nm. The luminous transmittance coefficients of IR filters (see Table 3) did not differ significantly for the following illuminant distributions: D65, A, and R1–R4. Similarly, as in the above-mentioned welding filters, in the case of illuminant R5 (room lit by radiation emitted from a laboratory furnace) the luminous transmittance coefficients (*τ_v/_*_day/R5_) for all the studied IR filters were lower as compared to other illuminants.

Relative changes in the luminous transmittance for IR filters and IR filter-IOL systems were calculated on the basis of data from Table 3 (see Equation (5)). Relative changes in the luminous transmittance for the tested IR filters (samples 9 to 13), determined for illuminant D65 vs. R5 amounted to 11.6%, 16.8%, 19.2%, 29.9%, and 7.14%.

The results are presented in Table 8 and Table 9 for IOLs with and without yellow chromophore, respectively. As can be seen from the tables, relative changes in the luminous transmittance were unaffected by the shade number of the filter. The differences between the various illuminant distributions were generally small, but it should be noted that they were slightly greater for IR filter-IOL systems without chromophore.

As can be seen from Table 10 and Table 11, the calculated transmittance reduction factors ranged from 0.88 to 0.99 relative units for all welding filter-IOL systems irrespective of the presence or absence of yellow chromophore in the IOL or filter characteristics. This indicates that the quality of vision through the analyzed filters and IOLs will not be significantly affected under the studied light conditions.

It was found that the type of illuminant had a significant effect on welding filters irrespective of IOL type. Transmittance coefficients calculated on the basis of the spectral characteristics of the standard illuminant D65 were compared with real illuminants imitating, e.g., poor light conditions in the workplace (illuminant R5). Significant changes for those coefficients (at 32%) were recorded for sample 8. In practice, this may affect the worker’s vision, compromising work comfort and quality. Similar results were found for IR filters used in the foundry industry. Relative changes in transmittance calculated for illuminant R5 as compared to the standard illuminant D_65_ were almost 30% (sample 12). In the case of welding filter-IOL systems, changes in luminous transmittance did not depend on the filter shade number. It was also found that in conjunction with welding filters, IOL type affects relative luminous transmittance. The systems containing IOLs without chromophore revealed higher levels of that coefficient.

## 5. Conclusions

The presented results show that analysis of the performance of filters used in conjunction with IOLs should include the transmittance reduction factor for filter-IOL systems, which is critical to preserving the protective characteristics of filters while ensuring good vision properties. It should be noted that analysis including merely luminous transmittance coefficients, which is applied in spectrophotometric research, may be insufficient for filter-IOL systems. The proposed testing methodology can be used for filter-IOL systems consisting of any type of protective optical filters and IOLs. The determination of light transmittance reduction factors is useful in that it can assist in the selection of optimal protective optical filters for people with implanted IOLs.

## Figures and Tables

**Table 1:** Luminous transmittance of intraocular lenses (IOLs) with and without yellow chromophore.

Sample no.	Sample Name	Luminous Transmittance (%)							Mean	SD
		Standard Illuminants (D65, A)		Real Illuminants (R1, R2, R3, R4, R5)						
		*τ_v/_*_day/D65_	*τ_v_*_/day/A_	*τ_v_*_/day/R1_	*τ_v_*_/day/R2_	*τ_v_*_/day/R3_	*τ_v_*_/day/R4_	*τ_v_*_/day/R5_		
1	Alcon SN6AT3	96.19	97.47	86.17	97.72	97.52	97.12	98.31	95.79	4.29
2	Artis PL E	95.60	95.89	95.46	95.88	97.73	95.68	96.22	96.01	0.65
***τ_v_*_/IOL/clear_ − *τ_v_*_/IOL/yellow_**		**−0.59**	**−1.58**	**9.29**	**−1.84**	**−0.15**	**−1.44**	**−2.09**	**0.23**	**4.05**
**((*τ_v_*_/IOL/clear_ − *τ_v_*_/IOL/yellow_)/*τ_v_*_/IOL/clear_) · 100%**		**−0.61**	**−1.62**	**10.78**	**−1.88**	**−0.15**	**−1.48**	**−2.13**	**0.44**	**4.62**

Notes: 1—IOL with yellow chromophore, 2—IOL without yellow chromophore, (Δ *τ_v_*_/IOL_ = *τ_v_*_/IOL/clear_ *τ_v_*_/IOL/yellow_)—difference in luminous transmittance between IOLs without and with yellow chromophore, (((Δ *τ_v_*_/IOL_ = *τ_v_*_/IOL/clear_ − *τ_v_*_/IOL/yellow_)/*τ_v_*_/IOL/clear_) · 100%)—relative changes in luminous transmittance between IOLs without and with yellow chromophore, SD—standard deviation.

**Table 2:** Luminous transmittance of welding filters (samples no. 3–8) and welding filter-IOL systems (for IOLs with and without yellow chromophore).

Sample no.	Luminous Transmittance (%)								
	Standard Illuminants (D65, A)		Real Illuminants (R1, R2, R3, R4, R5)					Mean	SD
	*τ_v/_*_day/D65_	*τ_v_*_/day/A_	*τ_v_*_/day/R1_	*τ_v_*_/day/R2_	*τ_v_*_/day/R3_	*τ_v_*_/day/R4_	*τ_v_*_/day/R5_		
**3**	**82.11**	**79.36**	**84.46**	**80.17**	**83.89**	**82.78**	**74.18**	**80.99**	**3.53**
3/1	79.17	77.34	81.37	78.30	81.83	80.42	72.87	78.76	3.06
3/2	78.46	76.03	80.06	76.81	80.27	79.19	71.3	77.45	3.12
**4**	**7.26**	**6.89**	**7.63**	**7.03**	**7.49**	**7.38**	**6.16**	**7.12**	**0.49**
4/1	7.02	6.73	7.37	6.87	7.31	7.17	6.05	6.931	0.45
4/2	6.94	6.61	7.28	6.73	7.17	7.06	5.92	6.726	0.68
**5**	**0.229**	**0.204**	**0.253**	**0.209**	**0.241**	**0.233**	**0.158**	**0.218**	**0.03**
5/1	0.222	0.198	0.244	0.204	0.235	0.226	0.155	0.212	0.03
5/2	0.219	0.195	0.242	0.200	0.230	0.222	0.152	0.209	0.04
**6**	**0.120**	**0.107**	**0.132**	**0.110**	**0.122**	**0.121**	**0.086**	**0.114**	**0.02**
6/1	0.116	0.105	0.127	0.108	0.120	0.118	0.084	0.111	0.01
6/2	0.114	0.103	0.125	0.106	0.117	0.116	0.082	0.109	0.01
**7**	**0.0432**	**0.0417**	**0.0471**	**0.0433**	**0.0427**	**0.0449**	**0.0366**	**0.0428**	**0.0032**
7/1	0.0420	0.0408	0.0458	0.0423	0.0417	0.0438	0.0359	0.0418	0.0031
7/2	0.0413	0.0399	0.0449	0.0415	0.0408	0.0429	0.0351	0.0409	0.0030
**8**	**0.0060**	**0.0053**	**0.0068**	**0.0055**	**0.0058**	**0.0061**	**0.0041**	**0.0057**	**0.0008**
8/1	0.0058	0.00518	0.00654	0.00536	0.0058	0.0059	0.0040	0.0055	0.0008
8/2	0.0057	0.00510	0.00650	0.00526	0.0056	0.0058	0.0039	0.0054	0.0008

Note: <sample no.>/1—welding filter-IOL system (for IOLs with yellow chromophore), <sample no.>/2—welding filter-IOL system (for IOLs without yellow chromophore), SD—standard deviation.

**Table 3:** Luminous transmittance of IR filters (samples no. 9–13) and IR filter-IOL systems (for IOLs with and without yellow chromophore).

Sample no.	Luminous Transmittance (%)								
	Standard Illuminants (D65, A)		Real Illuminants (R1, R2, R3, R4, R5)					Mean	SD
	*τ_v/_*_day/D65_	*τ_v_*_/day/A_	*τ_v_*_/day/R1_	*τ_v_*_/day/R2_	*τ_v_*_/day/R3_	*τ_v_*_/day/R4_	*τ_v_*_/day/R5_		
**9**	**38.58**	**36.37**	**40.46**	**36.71**	**38.30**	**38.59**	**32.93**	**37.42**	**2.40**
9/1	37.15	35.41	38.93	35.82	37.33	37.44	32.34	36.35	2.11
9/2	36.85	34.83	38.59	35.16	36.64	36.89	31.65	35.80	2.21
**10**	**29.56**	**27.56**	**31.21**	**27.76**	**29.56**	**29.49**	**24.58**	**28.53**	**2.14**
10/1	28.45	26.82	30.00	27.07	28.80	28.60	24.13	27.70	1.91
10/2	28.23	26.39	29.76	26.58	28.27	28.18	23.62	27.29	1.98
**11**	**3.02**	**2.70**	**3.30**	**2.73**	**2.83**	**2.30**	**2.44**	**2.86**	**0.28**
11/1	2.90	2.63	3.17	2.66	2.76	2.90	2.20	2.75	0.30
11/2	2.88	2.58	3.14	2.61	2.71	2.86	2.15	2.70	0.31
**12**	**2.34**	**2.37**	**2.32**	**2.43**	**2.58**	**2.42**	**2.27**	**2.39**	**0.10**
12/1	2.27	2.31	2.25	2.38	2.52	2.38	2.23	2.33	0.10
12/2	2.24	2.27	2.22	2.33	2.47	2.34	2.18	2.29	0.10
**13**	**0.84**	**0.85**	**0.87**	**0.88**	**0.89**	**0.89**	**0.78**	**0.86**	**0.04**
13/1	0.83	0.83	0.84	0.87	0.88	0.87	0.77	0.84	0.04
13/2	0.81	0.81	0.83	0.85	0.86	0.85	0.75	0.82	0.04

Note: <sample no.>/1—IR filter-IOL system (for IOLs with yellow chromophore), <sample no.>/2—IR filter-IOL system (for IOLs without yellow chromophore), SD—standard deviation.

**Table 4:** Relative changes in luminous transmittance determined for welding filters and welding filter-IOL systems (for IOLs with yellow chromophore).

Sample no.	Relative Change in Luminous Transmittance for Different Illuminants (%)							Mean (%)	Standard Deviation (%)
	D65	A	R1	R2	R3	R4	R5		
3	3.58	2.55	3.66	2.33	2.46	2.85	1.77	2.74	0.68
4	3.31	2.32	3.41	2.28	2.40	2.85	1.79	2.62	0.59
5	3.06	2.94	3.56	2.39	2.49	3.00	1.90	2.76	0.54
6	3.34	2.24	3.79	2.09	1.72	2.48	1,99	2.52	0.76
7	2.78	2.16	2.76	2.31	2.34	2.45	1.91	2.39	0.31
8	3.49	2.63	3.54	2.55	0.86	2.96	1.95	2.57	0.94

**Table 5:** Relative changes in luminous transmittance determined for welding filters and welding filter-IOL systems (for IOLs without yellow chromophore).

Sample no.	Relative Change in Luminous Transmittance for Different Illuminants (%)							Mean (%)	Standard Deviation (%)
	D65	A	R1	R2	R3	R4	R5		
3	4.45	4.20	5.21	4.19	4.32	4.34	3.88	4.37	0.41
4	4.41	4.06	4.59	4.27	4.27	4.34	3.90	4.26	0.23
5	4.37	4.41	4.35	4.31	4.56	4.72	3.80	4.35	0.29
6	4.76	3.74	5.30	3.64	4.10	4.13	4.09	4.52	0.59
7	4.40	4.32	4.67	4.16	4.45	4.45	4.10	4.36	0.19
8	4.49	4.14	4.13	4.36	4.30	4.61	4.14	4.31	0.19

**Table 6:** Transmittance reduction factors determined for welding filter-IOL systems (for IOLs with yellow chromophore).

Sample no.	Transmittance Reduction Factors for Different Illuminants (Relative Units)							Mean (%)	Standard Deviation (%)
	D65	A	R1	R2	R3	R4	R5		
3	0.96	0.97	0.96	0.98	0.98	0.97	0.98	0.97	0.01
4	0.97	0.98	0.97	0.98	0.98	0.97	0.98	0.97	0.00
5	0.97	0.97	0.96	0.98	0.98	0.97	0.98	0.97	0.01
6	0.97	0.98	0.96	0.98	0.98	0.98	0.98	0.97	0.01
7	0.97	0.98	0.97	0.98	0.98	0.98	0.98	0.98	0.00
8	0,97	0.97	0.96	0.97	0.99	0.97	0.98	0.97	0.01

**Table 7:** Transmittance reduction factors determined for welding filter-IOL systems (for IOLs without yellow chromophore).

Sample no.	Transmittance Reduction Factors for Different Illuminants (Relative Units)							Mean (%)	Standard Deviation (%)
	D65	A	R1	R2	R3	R4	R5		
3	0.96	0.96	0.95	0.96	0.96	0.96	0.96	0.96	0.00
4	0.96	0.96	0.95	0.96	0.96	0.96	0.96	0.96	0.00
5	0.96	0.96	0.96	0.96	0.95	0.95	0.96	0.96	0.00
6	0.95	0.96	0.95	0.96	0.96	0.96	0.96	0.96	0.01
7	0.96	0.96	0.95	0.96	0.96	0.96	0.96	0.96	0.00
8	0,96	0.96	0.96	0.96	0.96	0.95	0.96	0.96	0.00

**Table 8:** Relative changes in luminous transmittance determined for IR filters and IR filter-IOL systems (for IOLs with yellow chromophore).

Sample no.	Relative Change of Luminous Transmittance for Different Illuminants (%)							Mean (%)	Standard Deviation (%)
	D65	A	R1	R2	R3	R4	R5		
9	3.71	2.64	3.78	2.42	2.53	2.98	1.79	2.84	0.71
10	3.76	269	3.88	2.49	2.57	3.02	1.83	2.90	0.73
11	3.97	2.59	3.94	2.56	2.47	3.33	9.84	4.10	2.61
12	2.99	2.53	3.02	2.06	2.33	1.65	1.76	2.33	0.55
13	1.19	2.35	3.45	1.14	1.12	2.25	1.28	1.83	0.89

**Table 9:** Relative changes in luminous transmittance determined for IR filters and IR filter-IOL systems (for IOL without yellow chromophore).

Sample no.	Relative Change of Luminous Transmittance for Different Illuminants (%)							Mean (%)	Standard Deviation (%)
	D65	A	R1	R2	R3	R4	R5		
9	4.48	4.23	4.62	4.22	4.33	4.41	3.89	4.31	0.23
10	4.50	4.25	4.66	4.25	4.36	4.44	3.91	4.34	0.24
11	4.64	4.44	4.85	4.40	4.24	4.67	11.9	4.42	0.27
12	4.27	4.22	4.31	4.12	4.26	3.31	3.96	4.07	0.35
13	3.57	4.71	4.60	3.41	3.37	4.49	3.85	4.00	0.58

**Table 10:** Transmittance reduction factors determined for IR filter-IOL systems (for IOLs with yellow chromophore).

Sample no.	Transmittance Reduction Factors for Different Illuminants (Relative Units)							Mean (%)	Standard Deviation (%)
	D65	A	R1	R2	R3	R4	R5		
9	0.96	0.97	0.96	0.98	0.97	0.97	0.98	0.97	0.01
10	0.96	0.97	0.96	0.98	0.97	0.97	0.98	0.97	0.01
11	0.96	0.97	0.96	0.97	0.98	0.97	0.90	0.96	0.03
12	0.97	0.97	0.97	0.98	0.98	0.98	0.98	0.98	0.00
13	0.99	0.98	0.97	0.99	0.99	0.98	0.99	0.98	0.00

**Table 11:** Transmittance reduction factors determined for IR filter-IOL systems (for IOLs without yellow chromophore).

Sample no.	Transmittance Reduction Factors for Different Illuminants (Relative Units)							Mean (%)	Standard Deviation (%)
	D65	A	R1	R2	R3	R4	R5		
9	0.96	0.96	0.95	0.96	0.96	0.96	0.96	0.96	0.00
10	0.96	0.96	0.95	0.96	0.96	0.96	0.96	0.96	0.00
11	0.95	0.96	0.95	0.96	0.96	0.95	0.88	0.94	0.03
12	0.96	0.96	0.96	0.96	0.96	0.97	0.96	0.96	0.00
13	0.96	0.95	0.95	0.97	0.97	0.96	0.96	0.96	0.00

## Data Availability

The data presented in this study are available on request from the corresponding author.

## References

[1] Kernt M., Hirneiss C., Neubauer A.S., Lackerbauer C.A., Eibl K.H., Wolf A., Ulbig M.W., Kampik A. (2010). Protective effect of blue light-absorbing IOLs on the human retinal pigment epithelium. Ophthalmologe.

[2] Edwards K.H., Gibson A. (2010). Intraocular lens short wavelength light filtering. Clin. Exp. Optom..

[3] Eberhard R., Roberti P., Prünte C. (2009). Intraindividual comparison of color perception and contrast sensitivity with and without a blue light-filtering intraocular lens. Visual function with conventional and blue light-filtering IOLs. Eur. J. Ophthalmol..

[4] Wirtitsch M.G., Schmidinger G., Prskavec M., Rubey M., Skorpik F., Heinze G., Findl O., Karnik N. (2009). Influence of blue-light-filtering intraocular lenses on color perception and contrast acuity. Ophthalmology.

[5] Ardjomand N., Wirtitsch M.G., Wenzl E. (2009). Yellow intraocular lenses—To block or not to block. Eur. Ophthalmic Rev..

[6] Ao M., Chen X., Huang C., Li X., Hou Z., Chen X., Zhang C., Wang W. (2010). Color discrimination by patients with different types of light-filtering intraocular lenses. J. Cataract. Refract. Surg..

[7] Wang H., Wang J., Fan W., Wang W. (2010). Comparison of photochromic, yellow, and clear intraocular lenses in human eyes under photopic and mesopic lighting conditions. J. Cataract. Refract. Surg..

[8] Neumaier-Ammerer B., Felke S., Hagen S., Haas P., Zeiler F., Mauler H., Binder S. (2010). Comparison of visual performance with blue light–filtering and ultraviolet light–filtering intraocular lenses. J. Cataract. Refract. Surg..

[9] Mainster M.A. (2006). Violet and blue light blocking intraocular lenses. Photoprotection versus photoreception. Br. J. Ophthalmol..

[10] Mainster M.A., Turner P.L. (2010). Blue-blocking IOLs decrease photoreception without providing significant photoreception. Surv. Ophthalmol..

[11] Li X., Kelly D., Nolan J.M., Dennison J.L., Beatty S. (2017). The evidence informing the surgeon’s selection of intraocular lens on the basis of light transmittance properties. Eye.

[12] (2002). Personal Eye-Protection—Filters for Welding and Related Techniques—Transmittance Requirements and Recommended Use.

[13] (2002). Personal Eye-Protection—Infrared Filters—Transmittance Requirements and Recommended Use.

[14] Baszczyński K., Jachowicz M., Owczarek G., Szkudlarek J., Majchrzycka K. (2020). Head, Eye and Face Personal Protective Equipment. New Trends, Practice and Applications; Occupational Safety, Health, and Ergonomics: Theory and Practice.

[15] Crawford B.H. (1951). The scotopic visibility function. Proc. Phys. Soc. B.

[16] (1999). CIE Standard Illuminants for Colorimetry.

